# Radiation dose escalation by simultaneous modulated accelerated radiotherapy combined with chemotherapy for esophageal cancer: a phase II study

**DOI:** 10.18632/oncotarget.8050

**Published:** 2016-03-14

**Authors:** Jianzhou Chen, Hong Guo, Tiantian Zhai, Daniel Chang, Zhijian Chen, Ruihong Huang, Wuzhe Zhang, Kun Lin, Longjia Guo, Mingzhen Zhou, Dongsheng Li, Derui Li, Chuangzhen Chen

**Affiliations:** ^1^ Department of Radiation Oncology, Cancer Hospital of Shantou University Medical College, Shantou, Guangdong, China; ^2^ CRUK/MRC Oxford Institute for Radiation Oncology, University of Oxford, Oxford, United Kingdom; ^3^ Department of Radiation Oncology, Stanford University, Stanford, CA, USA; ^4^ Department of Oncology, The University of Hongkong - Shenzhen Hospital, Shenzhen, Guangdong, China; ^5^ Department of Public Health and Preventive Medicine, Shantou University Medical College, Shantou, Guangdong, China

**Keywords:** esophageal cancer, radiation therapy, dose escalation, simultaneous modulated accelerated radiotherapy, simultaneous integrated boost

## Abstract

The outcomes for patients with esophageal cancer (EC) underwent standard-dose radical radiotherapy were still disappointing. This phase II study investigated the feasibility, safety and efficacy of radiation dose escalation using simultaneous modulated accelerated radiotherapy (SMART) combined with chemotherapy in 60 EC patients. Radiotherapy consisted of 66Gy at 2.2 Gy/fraction to the gross tumor and 54Gy at 1.8 Gy/fraction to subclinical diseases simultaneously. Chemotherapy including cisplatin and 5fluorouracil were administered to all patients during and after radiotherapy. The data showed that the majority of patients (98.3%) completed the whole course of radiotherapy and concurrent chemotherapy. The most common ≥ grade 3 acute toxicities were neutropenia (16.7%), followed by esophagitis (6.7%) and thrombopenia (5.0%). With a median follow-up of 24 months (5-38) for all patients and 30 months (18-38) for those still alive, 11 patients (18.3%) developed ≥ Grade 3 late toxicities and 2 (3.3%) of them died subsequently due to esophageal hemorrhage. The 1- and 2-year local-regional control, distant metastasis-free survival, disease-free survival and overall survival rates were 87.6% and 78.6%, 86.0% and 80.5%, 75.6% and 64.4%, 86.7% and 72.7%, respectively. SMART combined with concurrent chemotherapy is feasible in EC patients with tolerable acute toxicities. They showed a trend of significant improvements in local-regional control and overall survival. Further follow-up is needed to evaluate the late toxicities.

## INTRODUCTION

Esophageal cancer (EC) is one of the most deadliest malignancies. The current standard of nonsurgical treatment for this disease is 50 Gray (Gy) of radiotherapy (RT) in conventional fractionation with concurrent chemotherapy. This regimen has been established since the 1990s [1, 2], though 50% of patients still had local failure after treatment. The Intergroup 0123 trial (INT0123) was undertaken to investigate the potential benefits of higher RT dose: 64.8Gy *vs*. 50.4Gy [3]. However, long-term follow-up showed that it did not improve the 2-year overall survival (OS: 31% *vs*. 40%) and local-regional control rates (LRC: 46% *vs*. 48%). Therefore, to date, the standard treatment for EC is still 50 Gy of conventionally fractionated RT with concurrent chemotherapy.

Intensity modulated radiotherapy (IMRT) is a modern high-precision RT technique which can reduce the dose to organs at risk (OARs) while maintaining the tumor coverage. A retrospective study demonstrated that EC patients treated with IMRT had less non cancer-related deaths, better LRC and OS, compared with patients treated with three-dimensional conformal radiotherapy (3DCRT) [4]. Meanwhile, there are also some studies suggesting that positive correlation between RT dose and LRC may exist in EC patients [5, 6]. Thus, it is logic to assume that patients with EC may have better LRC if treated with RT dose escalation using modern delivery technique.

Simultaneous modulated accelerated radiotherapy (SMART), also known as simultaneous integrated boost (SIB), is a novel dose escalation technique with IMRT, by which different dose fractionation could be delivered to OARs and tumors simultaneously. The smaller fraction size (< 2Gy) in OARs helps further reduce the risk and severity of toxicities, while the larger fraction size (> 2Gy) in tumors results in shorter treatment time and higher biologically equivalent dose. It is well acknowledged that tumor clonogen proliferation during conventional RT is a significant factor responsible for local failure of squamous cell carcinoma (SCC) of the upper respiratory and digestive tracts [7, 8]. A shorter overall treatment time of RT has been shown to be more beneficial to the treatment of EC [9, 10]. Additionally, one study found that the estimated alpha/beta of EC was 4.9 Gy which was lower than commonly expected, suggesting that hypofractionated RT may be more biologically effective for EC [6].

Taken together, we hypothesized that it was feasible to perform RT dose escalation with modest hypofractionation in EC patients by taking advantage of the better sparing of OARs using SMART technique. This was first supported by a dosimetric study reported previously [11]. We then began a single-arm phase II trial in 2012 to investigate the application of SMART combined with concurrent chemotherapy in EC (Clinicaltrial.gov number, NCT01670409, and Chinese Clinical Research Registry number, ChiCTR-ONC-12002356). The feasibility, safety and efficacy of this regimen in 60 enrolled patients were summarized here.

## RESULTS

### Patient characteristics

Between August 2012 and April 2014, a total of 60 patients were enrolled in this phase II trial. Patient characteristics were listed in Table 1. The majority of patients (78.3%) had locally advanced diseases (T_3/4_). The percentage of patients with positive lymph nodes (LNs) was 61.7%. Among the 60 patients, 11 (18.3%) of them were in stage IV due to supraclavicular LN metastasis.

**Table 1:** Clinical characteristics of 60 EC patients who received SMART combined with chemotherapy

Characteristics		No.	%
Age		Median 62 years (45-73)	
Gender	Male	50	83.3
	Femal	10	16.7
T stage*	1	1	1.7
	2	12	20.0
	3	32	53.3
	4	15	25
N stage*	0	23	38.3
	1	37	61.7
M stage*	0	49	81.7
	1_a_	3	5.0
	1_b_	8	13.3
Clinical stage*	II_A_	18	30.0
	II_B_	4	6.7
	III	27	45.0
	IV_a_	3	5.0
	IV_b_	8	13.3
Lesion site	Cervical	4	6.7
	Upper thoracic	25	41.7
	Middle thoracic	31	51.7

Abbreviations: EC: esophageal cancer. SMART: simultaneous modulated accelerated radiotherapy.

* According to American Joint Committee on Cancer (AJCC) staging system 6^th^.

### Quality review of treatment plans

Dose-volume parameters for planning target volumes (PTVs) were summarized in Table 2 according to International Commission on Radiation Units & Measurements 83 report [12]. The median V_20_ for the lungs and D_mean_ for the heart were 15.3% (3.8∼32.2%) and 11.0 Gy (0-38.9Gy) respectively. Planning objectives were well met in all patients.

**Table 2:** Dose-volume parameters of PTVs for the 60 EC patients who received SMART combined with chemotherapy

Parameters Median (range)	PTV_66_	PTV_54_
Volume (cm^3^)	74.3 (13.5 - 212.0)	199.5 (97.5 - 750.7)
D_2_ (Gy)	69.5(67.5-71.5)	67.7(59.6-70.6)
D_98_ (Gy)	65.2(62.9-66.1)	53.1(50.4-55.7)
D_50_ (Gy)	68.1(66.8-69.9)	59.2(56.1-62.9)
HI	0.07(0.03-0.11)	0.24(0.11-0.30)
CI	0.81(0.63-1.2)	0.79(0.59-0.88)

Abbreviation: PTV: planning target volume. EC: esophageal cancer. SMART: simultaneous modulated accelerated radiotherapy. Dx was defined as the minimum dose to a specified target volume. *HI*: Homogeneity Index. *HI* = D_5_/D_95_. D_5_ and D_95_ were the minimum doses received by the hot 5% and cold 95% of PTV, respectively.

*CI*: Conformity Index. $CI=\frac{V_{T,\mathrm{ref}}}{V_{T}}\times \frac{V_{T,\mathrm{ref}}}{V_{\mathrm{ref}}}\cdot V_{T}$. *V_T_*: The target volume. *V_T,ref_*: The target volume covered by reference isodose. *V_ref_*: The total volume covered by reference isodose.

### Treatment completion

All patients finished the entire planned course of RT, except one (1.7%) who received 97% of the prescribed dose due to grade 4 thrombocytopenia. The numbers of chemotherapy cycle that the patients completed were as follows: 4 cycles, 47 cases (78.3%); 3 cycles, 6 cases (10.0 %); 2 cycles, 6 cases (10.0%) and 1 cycle, 1 case (1.7%). Fifty-nine patients (98.3%) finished at least the concurrent chemotherapy. The main reasons that patients did not receive the remaining chemotherapy included severe haematological toxicities, esophagitis and patient's refusal. There were no dose modifications of chemotherapy. One patient (1.7%) underwent exploratory thoracotomy plus gastrostomy after completion of RT and 2 cycles of concurrent chemotherapy based on his own decision.

### Acute toxicities

The majority of patients only experienced mild acute toxicities (Table 3). The most common ≥ grade 3 acute toxicities were neutropenia (16.7%), followed by esophagitis (6.7%) and thrombopenia (5.0%). No treatment-related death was documented in the first 3 months since the beginning of treatment.

**Table 3:** Acute toxicities of the 60 EC patients who received SMART combined with chemotherapy

Toxicities No. (%)	Grade3	Grade4	Grade5
Neutropenia	5 (8.3%)	5 (8.3%)	0
Esophagitis	4 (6.7%)	0	0
Thrombopenia	2 (3.3%)	1 (1.7%)	0
Nausea/Vomiting	2 (3.3%)	0	0
Anemia	2 (3.3%)	0	0
Others	0	0	0

Abbreviations: EC: esophageal cancer. SMART: simultaneous modulated accelerated radiotherapy.

* Graded by Common Terminology Criteria for Adverse Events Version 4.0.

### Late toxicities

The last follow-up was October 10, 2015. As of this writing, 17 of 60 (28.3%) patients have died. The median follow-up was 24 months (5-38 months) for all patients and 30 months (18-38 months) for those still alive. The follow-up rate was 100%. The percentages of late toxicities were summarized in Table 4. Eleven patients (18.3%) developed ≥ Grade 3 late toxicities: 9 patients (15.0%) at the esophagus and the other 2 (3.3%) at lungs. Esophageal ulcers/fistulas were all found within the radiation dose escalation region. Two patients (3.3%) died subsequently due to esophageal hemorrhage. No severe late toxicities of skin, heart, spinal cord, hematologic system and liver had been reported.

**Table 4:** Late toxicities of the 60 EC patients who received SMART combined with chemotherapy

Toxicities No. (%)	Grade3	Grade4	Grade5
Esophageal ulcer/fistula	5 (8.3%)	0	2 (3.3%)
Esophageal stricture	3 (5.0%)	0	0
Lung	2 (3.3%)	0	0
Others	0	0	0

Abbreviations: EC: esophageal cancer. SMART: simultaneous modulated accelerated radiotherapy.

### Failure patterns and survivals

**Figure 1:**
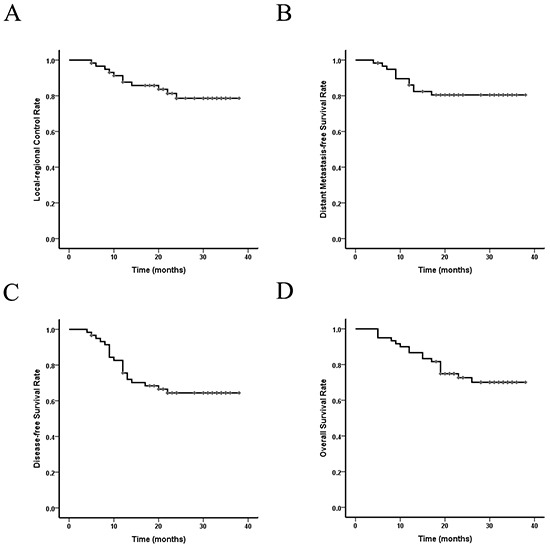
Local-regional control and survival curves of the 60 EC patients who received SMART combined with chemotherapy **A.** Local-regional control rate; **B.** Distant-metastasis free survival rate; **C.** Disease-free survival rate; **D.** Overall survival rate. Abbreviations: EC: esophageal cancer. SMART: simultaneous modulated accelerated radiotherapy.

There were no persistent diseases in all enrolled patients evaluated by contrast-enhanced computer tomography (CT) and barium swallow test. Within the follow-up time, tumor failure occurred in 20 patients (33.3%). The first site of failure was listed in Table 5. The most common pattern was distant metastasis (18.3%), followed by regional recurrence (11.7%) and local recurrence (8.3%). The LRC and survival curves were shown in Figure 1. The 1-year and 2-year LRC, distant metastasis-free survival (DMFS), disease-free survival (DFS), and OS rates were 87.6% and 78.6%, 86.0% and 80.5%, 75.6% and 64.4%, 86.7% and 72.7%, respectively.

**Table 5:** Failure patterns of 20 out of the 60 patients who received SMART combined with chemotherapy

First sites of failure	No	%
Distant metastasis	10	16.7
Regional recurrence	4	6.7
Local recurrence	4	6.7
Local and regional recurrence	1	1.7
Regional and distant	1	1.7

Abbreviations: EC: esophageal cancer. SMART: simultaneous modulated accelerated radiotherapy.

## DISCUSSION

Emerging dosimetric data suggests that RT dose escalation using modern delivery techniques may have a potential to improve the LRC of EC, however, the feasibility and efficacy of such approach have been unclear [11, 13, 14]. To our knowledge, this is the first report of phase II study of radiation dose escalation using simultaneous boost approach (SMART) for the treatment of EC. We found that it is feasible to apply SMART with concurrent chemotherapy in EC patients. Moreover, patients enrolled in this study showed a trend of significant improvements in LRC and OS.

The majority of patients in this study experienced mild acute toxicities and consistently, showed excellent compliance of treatment. Among the 60 patients, 98.3% and 78.3% of them finished the whole course of RT and chemotherapy, respectively, whereas only 67% and 66% of patients in the higher-dose arm of INT0123 trial completed RT and chemotherapy according to the protocol, respectively [3]. The treatment completion of our study is also comparable to patients underwent standard-dose RT using 3DCRT combined with different regimens of chemotherapy [15]. A couple of factors may contribute to the excellent treatment completion, including better sparing of OARs using SMART technique, lesser amount of 5Fluorouracil (5-Fu) administrated and smaller RT field.

In regard to late toxicities, we found that 18.3% of patients had ≥ Grade 3 late toxicities within the follow-up period. The rates of grade 4 and grade 5 toxicities were 0% and 3.3%, respectively, as compared to 8%and 2% in the combined modality arm of RTOG 85-01, and 24% and 10% in RTOG 92-07 [1, 16]. Thus, in comparison to histological data, our concurrent results do not suggest that SMART would significantly increase the risk of life-threatening toxicities and treatment-related death either. However, it should be noted that the follow-up in this study is relatively shorter than that in RTOG 85-01. Therefore, to more comprehensively evaluate the late toxicities, further follow-up is needed.

In line with the 3.3% of treatment-related death, patients enrolled in this study showed a trend of significant improvements in local tumor control and OS compared with previous studies, which is very striking. Only 8.4% of patient experienced local recurrences with a median follow-up of 30 months. The 2-year LRC and OS rates were 78.6% and 72.7%, respectively, as compared to 48% and 40% in the standard-dose arm of INT0123, despite more patients enrolled in this trial were in advanced stages:T_3/4_ stage (78.3% *vs.* 43%), N_1_ (61.7% *vs.* 17%) and M_1_ (18.3% *vs.* 0%) [3]. Apart from cancer stage, other factors, including target definition and radiation delivery technique, may also affect the outcomes of patients. The GTV in our study was determined by endoscopic ultrasonography (EUS), in addition to CT and esophageal barium swallow test that were used in INT0123, which may potentially reduce the risk of missing the target, resulting in better tumor control [17, 18]. However, the majority of local recurrences in INT0123 were actually within the GTV. Thus, it less likely that the improvement of tumor control in this study was derived from better target definition. Regarding the radiation delivery technique, IMRT and IGRT were applied in this study as compared to conventional RT technique in INT0123. Although advanced RT techniques could provide more accurate and precise treatment delivery, it is still unclear whether these advantages would lead to improvements in local tumor control and overall survival [4, 19–21]. Nevertheless, phase III trials comprising stand-dose arm using modern RT technique are warranted.

The dose escalation regimen applied in this trial was 66Gy in 2.2Gy/F. Whether further dose escalation is tolerable and beneficial to the patients is unclear. In a recently published phase I dose escalation study, Wen Yu, et al, suggested that it was feasible to deliver up to 62.5 Gy (2.5Gy/F) to GTV and 70Gy (2.8Gy/F) to the high risk region of GTV based on PET/CT, respectively, in EC patients [22]. However, the safety evaluation of dose escalation in this study relied mainly on acute toxicities without the information of late toxicities. Besides, in each dose escalation level, only 5 patients were recruited, whereas life-threatening esophagitis generally occurs in relatively smaller rate and requires larger sample size to evaluate. More importantly, of the 60 patients enrolled in our study, only 5 of them (8.4%) had local recurrence, while there were already 2 cases (3.3%) of treatment-related death due to esophageal hemorrhage. Thus, it is more likely that further dose escalation may lead to increased risk of treatment-related death, rather than better local tumor control.

One pitfall of this study is the lack of standard control. Comparison of SMART and standard-dose IMRT should be performed in future phase III trial. Additionally, all patients in this study were recruited in one single center. Whether this dose escalation regimen could be applied in different centers remains to be determined.

In summary, SMART combined with concurrent chemotherapy is feasible in EC patients with tolerable acute toxicities. They showed a trend of significant improvements in LRC and OS. Further follow-up is needed to evaluate the late toxicities. Phase III randomized trial is warranted to compare this regimen with standard-dose RT.

## MATERIALS AND METHODS

### Ethic statement

This study was in accordance with the Helsinki Declaration (2000) and was approved by the Clinical Research Ethics Review Committee of Cancer Hospital of Shantou University Medical College. Study-specific written informed consents were obtained from all patients prior to enrolment.

### Patient selection

Inclusion criteria were as follows: (a) pathologically proven primary esophageal SCC; (b) disease located in cervical, upper or middle thoracic esophagus; (c) no distant metastases (except supraclavicular lymph node); (d) Zubrod performance status: 0∼2; (e) adequate liver, renal and bone marrow function; (f) women of childbearing potential and male participants must practice adequate contraception.

Exclusion criteria included: (a) evidence of tracheoesophageal or mediastinal- esophageal fistula; (b) prior invasive malignancy (except non-melanomatous skin cancer) unless disease-free for a minimum of 2 years; (c) prior RT that would result in overlap of the planned RT fields; (d) severe and active comorbidities; (e) pregnant or nursing women.

### Pretreatment evaluation

The following evaluations were performed: medical history and physical examination, EUS of esophagus with biopsy, esophagography with barium swallow or iopromide (water-soluble nonionic contrast medium) when esophageal fistula may be present, plain and contrast-enhanced CT scan from the neck to the upper abdomen, abdominal ultrasound, electrocardiogram, hematologic and biochemical profiles. Bronchial endoscopies, bone scans and Positron emission tomography–CT (PET/CT) scans were performed as clinically indicated. All patients were staged using the American Joint Cancer Committee (AJCC) staging system 6^th^ [23].

### Radiotherapy

#### Immobilization and CT simulation

Patients were immobilized in supine position with the head and shoulders encompassed in a thermoplastic shell. Contrast-enhanced CT scan (3mm slice thickness) from the neck to the upper abdomen was obtained using a 16-slice CT scanner (The Philips Brilliance CT Big Bore Oncology Configuration, Cleveland, OH). CT images were then delivered to the Eclipse 10.0 treatment planning system (Varian Medical Systems, Palo Alto, CA) for target volume, OARs contouring and subsequent treatment planning.

#### Target volumes and prescribed doses

We have reported our treatment planning approach previously [11]. The gross tumor volume (GTV) includes the primary tumor (GTV_P_) of esophagus and positive regional lymph nodes (GTV_LN_). The contour of GTV was determined by CT images, endoscopic reports or barium/iopromide swallow fluoroscopy whichever larger. GTV_LN_ includes mediastinal or supraclavicular LNs if the shortest axis ≥ 1cm. Clinical target volume (CTV) was delineated with 2-cm longitudinal and 0.5- to 1.0-cm radial margins with respect to the GTV_P_ and a 0.5-cm uniform margin for GTV_LN_. Paraesophageal or tracheoesophageal groove LNs that did not meet the criteria of positive LN but their shortest axis ≥ 0.5cm were also encompassed in CTV. No other regional LNs were included in CTV for prophylactic irradiation. Two PTV_S_ were derived from the GTV and CTV, respectively: PTV_66_ = GTV + 0.5 cm and PTV_54_ = CTV + 0.5 cm. The prescribed dose was 66Gy/30F to PTV_66_ (2.2 Gy/F) and 54Gy/30F to PTV_54_ (1.8 Gy/F) in a single plan. OAR contours were created for the spinal cord, lungs, and heart.

#### Planning objectives

The planning objectives for PTV were 100% of the PTV volume receiving 95% of the prescribed dose. The dose constraints for OARs were as follows: spinal cord, D_max_ (maximum dose) < 45 Gy; heart, V_40_ (V_x_= percentage of the target volume receiving ≥ x Gy) < 100%, V_45_ < 67% and V_50_ < 33%; lungs, V_20_ < 30%, V_10_ < 50% and V_5_ < 60%.

#### Planning techniques

The SMART plans were generated using a sliding window dynamic delivery with 5 coplanar beams (angles: 210°/300°/0°/60°/150°). All plans were designed to be delivered using 6-MV photon beams from a linear accelerator (TrueBeam, Varian Medical Systems, Palo Alto, CA). Plans were optimized, selecting a maximum dose rate of 600 MU/min. Dose calculation was performed using Anisotropic Analytical Algorithm 8.6.02 with lung heterogeneity correction.

### Image-guided radiotherapy

Cone-beam CT (CBCT) scans were obtained in all patients prior to treatment delivery to assess potential setup errors at least once per week. Whenever the setup error is more than 5 mm in any dimension, surface markers on the thermoplastic shell that were used to align with radiation isocenter would be adjusted accordingly to correct the setup error. Adjustments would need to be validated in the next fraction of treatment. Re-simulation and re-planning would be considered if the setup error could not be corrected or the relationship between the tumor and adjacent critical structures changed significantly.

### Chemotherapy

All patients were treated with 2 cycles of concurrent chemotherapy on days 1 and 29, and 2 cycles of adjuvant chemotherapy on days 50 and 71. The chemotherapy regimen was as follows: cisplatin, 75 mg/m^2^, intravenous on day 1, 5-Fu 0.5 g/m^2^, intravenous drip infusion on day 1 to 4.

#### Dose modification

Dose modification of RT and chemotherapy was based on patient toxicities using common terminology criteria for adverse events (CTCAE 4.0) [24].

RT was delivered only when patients fulfilled the following conditions: (a) neutrophils ≥ 1.0×10^9^/L; (b) blood platelet ≥ 50×10^9^/L; (c) No grade 4 acute toxicities. For patients with grade 4 non-haematological toxicities, the remaining RT would be cancelled. RT would be suspended in patients with grade 3 non-haematological toxicities until they recovered. Patients could still receive treatment with ≤ grade 2 non-haematological toxicities unless further treatment would augment the severity of toxicities.

Chemotherapy was prescribed only when patients fulfilled the following conditions: (a) neutrophils ≥ 2.0×10^9^/L or ≥ 1.5×10^9^/L for appropriate patients without fever; (b) blood platelet ≥100×10^9^/L; (c) no grade ≥ 3 chemotherapy-related toxicities. For patients with grade 3 toxicities, chemotherapy should be postponed until the toxicities relieved. When patients experienced the first time of grade 4 bone marrow suppression, the dose of next cycle of chemotherapy would be reduced to 75% of the prescribed dose. The chemotherapy would be cancelled if patients had experienced twice of grade 4 bone marrow suppression. Carboplatin would be used to replace cisplatin if patients had significant nephro-, oto-, or neuro-toxicity.

### Assessment of acute toxicities

All patients were hospitalized during the treatment and assessed every week for acute toxicities using CTCAE 4.0. Routine evaluations included physical exam, hematologic and biochemical profiles, and esophagography (every 2 weeks during RT and once in each cycle of adjuvant chemotherapy).

### Follow-up

Patients were assessed at 3 months since the beginning of treatment for short-term efficacy, and then every 3 months for 2 years and every 6 months for 3 years. History, physical examination, hematologic and biochemical profiles, late toxicities assessment, chest X-ray plus esophagography or contrast-enhanced CT scan, and abdominal ultrasound were to be done at each visit. EUS of esophagus with biopsy or PET/CT was performed if clinically indicated. Local recurrence should be confirmed with pathological proof or at least two imaging examinations when biopsy was not applicable.

### Endpoints and sample size

The primary end points of this phase II trial were acute toxicities and 2-year late toxicities of esophagus and lungs. The secondary end points included 2-year LRC, DMFS, DFS and OS rates plus the first site of failure. In RTOG 85-01 trial, the 2-year LRC for patients underwent standard-dose RT and chemotherapy was approximately 50% [1]. Assuming the local control rate for patients enrolled in this study is 65%, 85 patients will be needed with an 80% power and 5% of potential early dropout or loss to follow-up. The toxicities and survival of the first 60 patients were summarized in this report.

### Statistical methods

Toxicities and survivals were measured from the beginning of treatment until the date of events (first site of failure or death) or the last clinic visit. The Statistical Package for Social Sciences (SPSS 21.0, Chicago, IL) was used to calculate the LRC, DMFS, DFS and OS using the Kaplan–Meier method.
